# Association of Tinnitus and Electromagnetic Hypersensitivity: Hints for a Shared Pathophysiology?

**DOI:** 10.1371/journal.pone.0005026

**Published:** 2009-03-27

**Authors:** Michael Landgrebe, Ulrich Frick, Simone Hauser, Goeran Hajak, Berthold Langguth

**Affiliations:** 1 Department of Psychiatry, Psychosomatics, and Psychotherapy, University of Regensburg, Regensburg, Germany; 2 Carinthia University of Applied Sciences, Feldkirchen, Austria; University of Sydney, Australia

## Abstract

**Background:**

Tinnitus is a frequent condition with high morbidity and impairment in quality of life. The pathophysiology is still incompletely understood. Electromagnetic fields are discussed to be involved in the multi-factorial pathogenesis of tinnitus, but data proofing this relationship are very limited. Potential health hazards of electromagnetic fields (EMF) have been under discussion for long. Especially, individuals claiming themselves to be electromagnetic hypersensitive suffer from a variety of unspecific symptoms, which they attribute to EMF-exposure. The aim of the study was to elucidate the relationship between EMF-exposure, electromagnetic hypersensitivity and tinnitus using a case-control design.

**Methodology:**

Tinnitus occurrence and tinnitus severity were assessed by questionnaires in 89 electromagnetic hypersensitive patients and 107 controls matched for age-, gender, living surroundings and workplace. Using a logistic regression approach, potential risk factors for the development of tinnitus were evaluated.

**Findings:**

Tinnitus was significantly more frequent in the electromagnetic hypersensitive group (50.72% vs. 17.5%) whereas tinnitus duration and severity did not differ between groups. Electromagnetic hypersensitivity and tinnitus were independent risk factors for sleep disturbances. However, measures of individual EMF-exposure like e.g. cell phone use did not show any association with tinnitus.

**Conclusions:**

Our data indicate that tinnitus is associated with subjective electromagnetic hypersensitivity. An individual vulnerability probably due to an over activated cortical distress network seems to be responsible for, both, electromagnetic hypersensitivity and tinnitus. Hence, therapeutic efforts should focus on treatment strategies (e.g. cognitive behavioral therapy) aiming at normalizing this dysfunctional distress network.

## Introduction

Tinnitus, the perception of sound in the absence of an external sound, is a frequent disorder of auditory perception, which is very difficult to treat [1]. Tinnitus as a phantom perception of a meaningless sound has to be differentiated from auditory hallucinations which mainly occur in the context of psychiatric diseases and are characterized by e.g. the perception of voices. About 10–20% of the adult population experiences some degree of tinnitus. Many learn to ignore the sounds and experience no major effects, but for about 1 in 100 adults, the noise interferes significantly with daily life [2]. In those patients, tinnitus is frequently associated with neuropsychiatric co-morbidity such as depression, anxiety or sleep disorders [3], [4], which underlines the clinical and socio-oeconomic importance.

Even if the pathophysiology of tinnitus remains incompletely understood, there is growing evidence that dysfunctional neuroplastic processes in the brain are involved. In particular, it is assumed that tinnitus might be the correlate of maladaptive neuroplastic changes due to distorted sensory input [5], [6]. Accordingly functional imaging studies demonstrated neuroplastic alterations in the central auditory system [7], [8]. However tinnitus related alterations of neural functioning are not limited to the central auditory system, but also encompass non-auditory regions such as frontal and limbic areas [9]–[12].

There has been an ongoing debate, whether tinnitus might be related to exposure to electromagnetic fields (EMF) [13]. One previous study found a tinnitus prevalence of 14% in a sample of electromagnetic hypersensitive subjects [14]. Whereas electromagnetic hypersensitivity per se is not a proxy variable for EMF-exposure, substantial evidence from electrophysiological studies has shown EMF and especially mobile phone emissions to influence cognitive function [15] and neuronal processing in the central auditory system [16]–[20]. These might represent potential mechanisms by which EMF could contribute to the development of tinnitus. However, two recent epidemiological studies from a student and a the general population, respectively, did not demonstrate a significant relationship between mobile phone use and tinnitus [21], [22].

Besides the hypothesized involvement in the generation of tinnitus, EMF-exposure has also been related to a variety of unspecific health symptoms (e.g., dizziness, fatigue, headache, sleep disturbances, etc.). Despite a huge amount of studies investigating the health impact of EMF, no clear relationship between EMF-exposure and these unspecific health symptoms could be established and the majority of provocation studies failed to demonstrate such a relationship [23]. Based on the fact that some individuals suffer from a variety of symptoms, which they attribute to EMF-exposure, whereas the overwhelming majority does not experience any symptoms under the same EMF-exposure, the concept of “subjective electromagnetic hypersensitivity” evolved [24]. This subjective electromagnetic hypersensitivity is characterized by health complaints, which interfere with daily living and are subjectively attributed to electromagnetic fields of named emission sources (e.g., mobile phone base stations, hot spots, TV-sets, etc.). Very recent data from an epidemiological case-control study suggest that this subjective electromagnetic hypersensitivity is characterized by dysfunctional cognitions, reduced discrimination ability for sensory stimuli [25] and increased sensitivity of a cortical network encompassing the anterior cingulate and insular cortex [26].

Due to the large sample size, the detailed clinical and neurobiological characterization and the control group, which was matched for age, gender and either living surroundings or workplace (as very rough proxies for EMF-exposure), this study population [25] was well suited to investigate the relationship between tinnitus, subjective electromagnetic hypersensitivity and EMF-exposure. In detail, we addressed the following questions: 1.) Do subjective electromagnetic hypersensitive people suffer more often from tinnitus than controls? 2.) Are there clinical characteristics that point to potential common pathological mechanisms?

## Materials and Methods

### Ethics statement

The study has been approved by the local ethics committee of the University of Regensburg. All participants gave written informed consent.

### Study population

Tinnitus occurrence and severity have been assessed in a sample of subjects who claimed themselves to be hypersensitive to electromagnetic fields (EMF-sensitive). Subjective electromagnetic hypersensitivity was defined by the occurrence of unspecific health complaints interfering with daily living and the subjective belief that these complaints are caused by named electromagnetic emission sources (e.g., mobile phone base stations, hot spots, TV-sets, etc.) [25]. This EMF-sensitive group was compared to an age- and gender-matched control sample, which were living in the same close vicinity or working at the same workplace in a comparable position (1∶2 matching if the patient was working, 1∶1 if not working) but not expressing the subjective belief to be electromagnetic hypersensitive. This matching procedure should minimize potential influences of environmental physical (EMF-exposure) and social stressors. The sample of electromagnetic hypersensitive patients (89 cases and 107 controls) was intensively characterized with both psychological and neurobiological measurements (e.g., discrimination ability and cortical excitability determined by transcranial magnetic stimulation, genetic polymorphisms of the serotonin transporter and the dopamine-d4-receptor) in addition to a detailed sociobiographic, medical and EMF-specific history. For details of recruitment and measurements taken, see Landgrebe et al. 2008 [25]. In brief, the symptom load of electromagnetic hypersensitive patients on a psychometric scale measuring intensity of various bodily complaints, cognitive and mood annoyances, and sleeping problems were measured. These symptoms despite their physiological heterogeneity share in common that they all were alleged by electromagnetic hypersensitive patients to be caused by EMF-exposure and that they formed a Rasch-conform homogeneous complaint score in the sense of a psychological trait. A score of at least 19 points on this “Regensburg-EMF-complaint-list”, which corresponds to the health complaint level of the upper terzile in the general population [27], was used as inclusion criterion. Further inclusion criteria were attribution of the health symptoms to named electromagnetic emission sources (e.g. mobile phone base stations, hotspots, etc.) and age between 18 and 75 years. Excluded were all patients suffering from conditions that precluded transcranial magnetic stimulation. 135 electromagnetic hypersensitive patients were screened for the study, 34 did either not fulfil inclusion criteria or had to be precluded due to the exclusion criterion. 89 of the 101 eligible electromagnetic hypersensitive patients finally agreed to participate. From the group of not employed electromagnetic hypersensitives, 30 controls living in the same surroundings had been nominated and 12 controls were contacted via random procedures. From the group of employed electromagnetic hypersensitives, all working place controls had been nominated (n = 27), controls living in the same surroundings had been nominated in 25 cases and were contacted at random in 13 cases. If the electromagnetic hypersensitive proband nominated more than one control from his/her living surroundings, all controls were asked for participation.

### Questionnaires

Tinnitus occurrence and duration in the study population were assessed by the following questions: (1.) Do you currently perceive tinnitus? (2.) If yes, since how long? Furthermore, in all tinnitus sufferers tinnitus severity was assessed by a German translation [28] of the Tinnitus Handicap Inventory (THI; [29]). Tinnitus was not an item of the Regensburg-EMF-complaint-list [27].

### Statistics

Screening procedures for potential selective non-response were performed using Chisquare statistics and t-tests. A multivariate model for risk of tinnitus was estimated by means of logistic regression using stepwise inclusion/exclusion of potential predictor variables (p_in_ = 0.05, p_out_ = 0.10) as model building strategy. The following variables were evaluated: (1) age at study entry; (2) subjective hypersensitivity to EMF, ability to differentiate electromagnetic evoked sensory stimuli, number of complaints in the Regensburg-EMF-complaint-list as an indicator of severity of subjective electromagnetic hypersensitivity; (3) gender; (4) measures of cortical excitability as determined by standard procedures with transcranial magnetic stimulation (i.e., active and resting motor thresholds, intracortical inhibition and facilitation, cortical silent period; for technical details see [25]; (5) global score of the Pittsburgh Sleep Quality Index (PSQI; [30]); (6) noise exposure or incremental EMF-exposure due to mobile phone use (approximated by the amount of the last invoice). These variables have been chosen because they either represent typical clinical features of electromagnetic hypersensitivity (e.g. 2) or represent known typical risk factors of or are associated with tinnitus (e.g. 1, 3, 5 and 6) or are known to be associated with typical psychological traits, which may also be associated with tinnitus or electromagnetic hypersensitivity (e.g. 4; [31]). Simultaneous estimation of the impact of electromagnetic hypersensitivity and tinnitus on sleep quality as well as on discrimination ability was performed via a linear regression model specifying the respective dummy-variables for potential predictors. All analyses were calculated via SAS statistical software.

The study has been approved by the local ethics committee of the University of Regensburg. All participants gave written informed consent.

## Results

The questionnaires were sent to all 196 participants of the original electromagnetic hypersensitivity study [25]. 77.5% (69 out 89) of the EMF-sensitive group and 74.8% (80 out of 107) of the control group returned completed questionnaires resulting in an overall response rate of 76% (149 out of 196). Non-responders did not differ from responders with respect to sex, age, education, employment situation, sleep quality, body mass index, utilization of the health system (estimated by the number of medical consultations), utilization of mobile phones, number of sick days, or EMF-symptom load assessed with the Regensburg-EMF-complaint-list. Study participants who at the time point of their interview had qualified for a diagnosis of major depression according to the WHO CIDI short form [32] sent back the tinnitus questionnaires only in 60%, whereas all other participants answered the questionnaire in 79% (Fisher's exact test: p = 0.026). In contrast, a co-morbid anxiety disorder or somatoform disorder according to WHO CIDI had no effect on the response rates. Taken together, the sample of electromagnetic hypersensitive patients and controls responding to the questionnaire was comparable with the whole study population.

All further analyses regard only the 149 responders. Major depression (22%; Chi-square: p = 0.0008), anxiety disorder (6%; Chi-square: p = 0.029) and somatoform disorders (9%; Chi-square: p = 0.0071) were significantly more frequent in the EMF-sensitive group compared to the control group (4%, 0%, 0%, respectively). Furthermore, electromagnetic hypersensitive patients had a higher EMF-complaint level and a worse sleep quality (table 1). Electromagnetic hypersensitive patients and controls did not differ with respect to age, weight, height and body mass index (table 1). Tinnitus was reported significantly more often in the EMF-sensitive group compared to the control group (50.72% vs. 17.5%; Chi-square p<0.0001) with no differences between both groups with respect to tinnitus duration and severity as assessed by the THI (table 1).

**Table 1:** Sociodemographic data, sleep quality, EMF-complaint score, and tinnitus duration and severity of electromagnetic hypersensitive patients and controls.

Group	EHS (N = 69)		Controls (N = 80)		Differences
	Mean	SD	Mean	SD	*P*-Value
**Age [years]**	50.4	±10.6	49.9	±10.6	0.81
**Proportion females**	56.5%		66.3%		
	**Mean**	**SD**	**Mean**	**SD**	
**Body mass index**	25.0	±4.2	25.2	±4.0	0.75
**Subjective sleep quality (PSQI)**	9.1	±3.1	6.4	±2.1	<0.0001
**EMF complaint score**	46.3	±21.4	13.7	±12.4	<0.001
**Subjects with tinnitus**	35		14		<0.0001
**Tinnitus Duration [months]** *	121.94	±124.43	107.36	±82.486	0.69
**THI** *	35.059	±23.87	22.923	±18.773	0.11

* : mean and standard deviation refer only to subjects with tinnitus

Using a logistic regression analysis the following four items were found to be independent predictors for tinnitus (table 2). These were (1.) subjective belief of being electromagnetic hypersensitive, (2.) male, (3.) reduced sleep quality as assessed by the PSQI, and (4.) reduced discrimination ability for electromagnetic evoked sensory stimuli. In contrast, a history of exposure to noise (p = 0.5187) as well as a high score on the Regensburg-EMF-complaint-list was not associated with the risk of suffering from tinnitus. Furthermore, incremental electromagnetic field exposure in addition to the one acquired in subjects' working and living environment was quantified by the degree of utilizing a mobile phone and could not be shown to influence the risk of tinnitus (p = 0.5116).

**Table 2:** Items increasing the probability to suffer from tinnitus.

Variable	Estimate	Error	Chi-Square	Pr>ChiSq
Intercept	1.9784	0.6458	9.3858	p = 0.0022
Being electromagnetic hypersensitive	−0.9838	0.4688	4.4046	0.0358
female	1.6906	0.4620	13.3889	0.0003
PSQI-score	−0.2470	0.0821	9.0418	0.0026
Discrimination ability (real from sham magnetic pulses)	0.0283	0.0115	6.0440	0.0140

The probability modelled is that for having no tinnitus, i.e. negative estimates increase the probability of having tinnitus. From all collected items, subjective belief to be electromagnetic hypersensitive, male gender, high PSQI-score (bad quality of sleep) and a low ability to discriminate real from sham magnetic pulses are significantly increasing the risk of suffering from tinnitus.

Since an association between tinnitus and sleep disorders is well known [3], we estimated to which extent the reduced sleep quality in the EMF-sensitive group is explained by the increased prevalence of tinnitus in this group by performing a linear regression analysis with the PSQI as the dependent variable. The interaction “group membership*tinnitus” proved not to be significant (p = 0.60) indicating that claiming oneself as electromagnetic hypersensitive and suffering from tinnitus are independent risk factors for sleep disturbances with electromagnetic hypersensitivity exerting an even greater influence on sleep quality than tinnitus (table 3).

**Table 3:** Estimation of the impact of being electromagnetic hypersensitive or having tinnitus on sleep quality.

Variable	Estimate	Error	T-value	Pr>ChiSq
Intercept	6.2209	0.2994	20.78	<0.0001
Being electromagnetic hypersensitive	2.2768	0.4548	5.01	<0.0001
Tinnitus	1.1664	0.4830	2.42	0.0170

The probability modelled is that for worsening sleep quality assessed by the PSQI, i.e. positive estimates increase the PSQI indicating worse sleep quality. Electromagnetic hypersensitivity and tinnitus are independent risk factors for bad sleep quality with electromagnetic hypersensitivity having a more severe effect than tinnitus.

Interestingly, a reduced ability to discriminate real from sham magnetic pulses, which is typically diminished in subjectively electromagnetic hypersensitive subjects [25], has been found to be an independent predictor of tinnitus. To further investigate the relationship between tinnitus and subjective electromagnetic hypersensitivity, we estimated to which extent this reduced discrimination ability is explained by the subjective belief of being electromagnetic hypersensitive and/or by suffering from tinnitus. For this purpose, we calculated exactly the same statistical ANOVA model for subjects' discrimination abilities within the subsample having answered the tinnitus questionnaire (i.e. all subjects experiencing tinnitus from the electromagnetic hypersensitive and the control group). This analysis revealed, irrespective of the diminished statistical power, the identical main and interaction effects as for the original sample [25]: People's ability to discriminate the stimuli was again depending on gender, age, subjective belief of being electromagnetic hypersensitive, sequence of stimulus presentation (sham/verum), and an interaction effect of age and subjective belief (all F>5; d.f. = 1, 141 each, p<0.02). Adding the total score of the Regensburg-EMF-complaint-list (a measure of unspecific health complaints) as a linear covariate to the statistical model yielded no additional information with respect to discrimination ability (F = 0.73; p = 0.39). But introducing a new classification variable (tinnitus being present or not) to the ANOVA improved the statistical model significantly: People suffering from tinnitus displayed a diminished ability to discriminate between a magnetic and a sham pulse (F = 4.21, d.f. = 1, 141; p = 0.042). This effect was independent from that one of their subjective belief of being electromagnetic hypersensitive. Both effects were in the same direction and did not interact with each other (F for interaction = 1.28; p = 0.260), their effect sizes were comparable.

## Discussion

The primary aim of this study was to elucidate whether a potential relationship exists between EMF-exposure, electromagnetic hypersensitivity and tinnitus. The main finding is that tinnitus is much more frequent in subjectively electromagnetic hypersensitive patients compared to control subjects. Independent predictors of tinnitus occurrence were subjective belief of being electromagnetic hypersensitive, being male, a reduced sleep quality, and a reduced ability to discriminate real from sham electromagnetic evoked sensory stimuli. In contrast, no evidence for a relationship between EMF-exposure and tinnitus has been found.

The observed prevalence of tinnitus of 17.5% in the control group is in accordance with findings from various epidemiological studies showing similar rates [33]. In addition, it is well known that tinnitus is more frequent in males than in females [33], which has been confirmed in our study sample. Furthermore, we found that reduced sleep quality, as reflected by a high PSQI score, represents an independent predictor for tinnitus, underscoring the well-established relationship between tinnitus and sleep problems [3]. So far, the results of this study confirm findings of other studies with respect to prevalence rates, co-morbid sleep disturbances and higher prevalence in males.

A new finding is the surprisingly high prevalence of tinnitus among the electromagnetic hypersensitive patients. Since the study design at least partially controlled for environmental EMF-exposure by recruiting patients and controls from the same private and working environments, the increased prevalence in the EMF-sensitive group can hardly be explained by differences in environmental EMF-exposure. Furthermore the utilization of mobile phones did not show any significant relationship to tinnitus. Our data thus indicate that the increased prevalence of tinnitus may rather be due to other factors raising the question about the nature of this relationship. One possibility is that tinnitus just represents another unspecific health symptom of subjectively electromagnetic hypersensitive patients. Another possibility is that tinnitus and subjective electromagnetic hypersensitivity share a common pathophysiology. A key feature repeatedly found in subjectively electromagnetic hypersensitive patients is reduced discrimination ability for magnetic pulses [25], [34], which has been shown in this study to be also an independent predictor of tinnitus. In addition, we could demonstrate that both tinnitus and electromagnetic hypersensitivity are independent predictors for reduced discrimination ability. These results suggest that tinnitus seems not to represent just one more symptom on a list of interchangeable complaints that electromagnetic hypersensitives suffer from. Instead, tinnitus and electromagnetic hypersensitivity may share pathophysiological similarities which are related to alterations in sensory discrimination.

### Pathophysiological considerations

With the failure to prove a causal relationship between EMF-exposure and symptoms in subjectively electromagnetic hypersensitive patients [23], research is focusing increasingly on neuronal mechanisms involved in symptom formation. Recent results suggest an individual vulnerability of these patients against environmental stressors especially affecting the autonomic nervous system [35]–[37]. A pilot study investigating possible alterations of central nervous system excitability found evidence for alterations of the glutamatergic system [38], which may be an indicator of reduced adaptation abilities of these patients. These results have been replicated in a larger study population [25] underlining the robustness of these findings. Furthermore, specific dysfunctional cognitions dealing with different aspects of EMF were identified to play a pivotal role in the generation of subjective electromagnetic hypersensitivity. The importance of these cognitive processes is supported by the efficacy of cognitive behavioral therapy for the treatment for electromagnetic hypersensitivity [39]. In addition, functional imaging revealed the involvement of anterior cingulate and insular cortex in symptom generation [26]. These areas, which are part of a neural network conveying distress and avoidance in pain perception [40], [41], seem also to play a pivotal role in subjective electromagnetic hypersensitivity [26] or other functional somatic syndromes like e.g. multiple chemical sensitivity [42]. With respect to tinnitus, the increased prevalence in electromagnetic hypersensitive patients could be due to the increased sensitivity of this cortical distress network, which has been repeatedly shown to be involved in the pathophysiology of tinnitus [9]–[12], [43], [44].

The dysfunctional over-activation of this cortical neural network might be related to a disturbed representation of external and internal perceptions, which in turn could explain the reduced ability to discriminate real from sham electromagnetically evoked stimuli of electromagnetic hypersensitive patients [25] as well as in subjects experiencing tinnitus.

Taken together these results point to a shared pathophysiology of subjective electromagnetic hypersensitivity and tinnitus. It may be hypothesized that these changes represent a key feature of somatoform disorders, which should be addressed in future studies.

Although this study thus provides some interesting new findings, it is not without limitations. First, data were cross-sectional and correlation analyses were used, which makes it difficult to determine the exact nature of the relationships between the variables of interest. Hence, prospective, longitudinal studies are needed to establish the precise nature and the directions of the relationships explored in this study. Second, self-report measures were used for tinnitus assessment. Such measures may not accurately capture the phenomena under investigation. Third, the study design aimed at minimizing the influence of environmental physical (e.g.; EMF-exposure) and social stressors. We are well aware that this design cannot guarantee equivalent EMF-exposure between both groups. However, it is noteworthy that the discrimination ability to differentiate real from sham magnetic stimuli, which was shown to be diminished in subjective electromagnetic hypersensitive patients and tinnitus sufferers, has been assessed under laboratory conditions in a double-blind, randomized design [25].

In conclusion, this study has shown that tinnitus is much more frequent among subjective electromagnetic hypersensitive patients whereas there is no hint for a relationship between tinnitus and exposure to electromagnetic fields. Rather, the correlation between tinnitus and electromagnetic hypersensitivity might be due to an individual vulnerability. Neurobiological characteristics of this increased vulnerability such as an oversensitive cortical distress network and an impaired discrimination ability for electromagnetically evoked sensory stimuli might be involved in the pathophysiology of both tinnitus and electromagnetic hypersensitivity and possibly also in other related perception disorders. Nevertheless, this hypothesis derived from our epidemiological study has to be confirmed in further studies by e.g. intervention studies aiming for a normalization of the postulated over-activated distress network in subjectively electromagnetic hypersensitive (e.g. cognitive behavioral therapy, which has been shown to be successful in electromagnetic hypersensitivity [45] and tinnitus patients).
